# Factors Influencing Mandibular Invasion, Lymph Node Metastasis and Extracapsular Spread in Squamous Cell Carcinoma of the Oral Cavity

**DOI:** 10.3390/cmtr18030030

**Published:** 2025-06-27

**Authors:** Rathindra Nath Bera, Richik Tripathi

**Affiliations:** Department of Oral and Maxillofacial Surgery, Faculty of Dental Sciences, Institute of Medical Sciences, Banaras Hindu University, Varanasi 221005, India; richik.tripathi@gmail.com

**Keywords:** pattern of invasion, worst pattern of invasion, ECS, lymph node metastasis, bone invasion

## Abstract

Background: A number of factors might affect survival in oral squamous cell carcinoma. Nodal status is one of the most important prognosticators affecting survival. Studies have shown that pattern of invasion is an important aspect related to survival. Study design: retrospective single-center study (original article). Objectives: Our study aimed at evaluating the factors affecting mandibular invasion, lymph node metastasis, and extracapsular spread in oral squamous cell carcinoma and the survival factors associated with it. Methods: Patient records were evaluated to identify factors influencing primary outcome and survival. Cox regression analysis and Kaplan Meir were applied to evaluate the outcomes. Youden’s index was used to determine a cut-off value for depth of invasion and lymph node size affecting outcome. A *p* value of <0.05 was considered statistically significant. Results: The study evaluated 162 patients with oral cancer. The cut-off value for DOI was 6.5 mm, significantly affecting mandibular invasion and cervical metastasis. The cut-off value for lymph node size was 2.95 cm, significantly affecting extracapsular spread and overall survival. An aggressive pattern of invasion significantly affects mandibular invasion, cervical metastasis, and survival. Conclusion: An aggressive pattern of invasion and depth of invasion are independent risk factors for cervical lymph node metastasis and mandibular invasion. The independent risk factor for extracapsular spread is lymph node size. Lymph node metastasis and nodal size, pattern of invasion, mandibular invasion, and extracapsular spread are independent risk factors affecting overall survival.

## 1. Introduction

Head and neck cancer is the third most common cancer in South Asia, and the majority of cases are oral squamous cell carcinomas [1]. The prognosis of oral squamous cell carcinoma (OSCC) is influenced by multiple factors, with the stage of the primary tumor and the nodal status being the most significant. Tumor size, grade, depth of invasion, perineural and lymphovascular invasion, tumor budding, worst pattern of invasion (WOPI), and lymphoid response are all risk factors for nodal metastasis [2,3]. Extracapsular spread (ECS) is one of the most important prognostic factors influencing survival in head and neck squamous cell carcinomas (HNSCCs). It may be present in up to 60% of patients with HNSCC, and microscopic ECS can be found in 10.5% to 25% of patients with a clinically N0 neck. Notably, ECS may also be present in metastatic lymph nodes measuring less than 2 mm in size. A number of factors might influence the presence of ECS: size and number of metastatic lymph nodes, contralateral neck metastasis, depth of invasion, size of primary tumor, etc. [4,5].

The POI can be divided into five categories; POI 1: pushing well-delineated infiltrative borders, POI 2: finger-like pushing pattern/infiltrative solid cords or bands and/or strands of cells, POI 3: small groups or cords of cells >15 cells, POI 4: small group of cells <15 or single cell, and POI 5: satellite tumor nodules >1 mm from the main tumor [6,7,8,9]. POI 5 was associated with poor prognosis in oral and oral pharyngeal cancers and in 2018 was added to the ICCR database as an obligatory element for reporting [10]. POI 5 has been reported to be an independent prognosticator associated with poor overall survival [11].

Bone invasion by oral squamous cell carcinoma (OSCC) is classified as T4a, indicating advanced head and neck cancer. The prognostic significance of mandibular invasion remains a subject of debate, possibly due to the lack of distinction between cortical and medullary involvement [12]. Mandibular involvement may be present in more than 50% of OSCC cases [13,14]. Marginal mandibulectomy is typically indicated when there is cortical erosion or periosteal involvement, whereas segmental mandibulectomy is preferred in cases of gross paramandibular spread or medullary invasion [15,16]. Our study aimed at evaluating the influence of various factors on mandibular invasion, lymph node metastasis, and extracapsular spread in oral squamous cell carcinoma.

## 2. Patients and Methods

### 2.1. Study Design and Setting

The following study was undertaken in a tertiary care center in an Indian institute of national importance, from January 2013 to December 2021. Ethical approval was obtained from the institute’s ethical committee (No. Dean/2021/EC/2696), and the study followed STROBE guidelines [17].

### 2.2. Participants

Medical records of the institute from January 2013 to December 2021 were evaluated. Patients who were operated on for OSCC and underwent mandibulectomy (marginal/segmental) were included in the study. Inadequate data and inability to review pathological reports or archive them were the exclusion criteria.

All records were evaluated by one senior surgeon, one pathologist, and one radiologist blinded to the outcome.

### 2.3. Evaluation and Outcome

From the medical records, data were obtained for clinical staging of the primary tumor, radiological investigations, histopathology, treatment of the neck, and adjuvant therapies. The clinical staging was performed on the basis of clinical examination, biopsy, CT/MRI, OPG for primary tumor, and CT/MRI/USG FNAC of the neck. Records obtained on the clinical staging of each patient were further updated according to the 8th edition of AJCC [9].

According to the archived records, patients with clinically N0 staging underwent selective neck dissection (SND) I–III for all primary sites except for oral tongue, for which SND I–IV was undertaken. Modified radical neck dissection (MRND) sparing internal jugular vein IJV (IJV) and sternocleidomastoid was performed for node-positive necks. Bilateral neck dissection was performed for anterior tongue tumors and anterior floor of mouth tumors. Adjuvant radiotherapy was given to patients with pathological positive nodes, higher T stage (T3/T4), lymphovascular and perineural invasion, positive margins, and ECS. Adjuvant chemoradiation was given to patients with positive margins and ECS. As initially described, POI was divided into five categories and broadly classified into two major categories: POI (1,2,3)-cohesive pattern and POI (4,5)-aggressive pattern. The five broad categories were; I: broad tumor front, II: finger-like tumor front, III: tumor islands >15 cells, IV: tumor islands <15 cells, and V: tumor islands with <15 cells more than 1 mm apart. ECS was determined from histological records as either present or absent. No data could be obtained on either ECS being microscopic or macroscopic. ECS was considered positive when there was an extension of tumor >2 mm beyond the lymph node capsule with or without stromal reaction. Mandibular invasion data were obtained from radiological records (CT) for either no involvement or cortical or medullary involvement. Similar records were also obtained from histological reports. The pattern of invasion into the mandible was also recorded a serosive or infiltrative. For statistical purposes, the histological data on mandibular invasion were taken into consideration. Depth of invasion (DOI) was recorded in accordance with the final histopathological reports. It was measured by drawing a perpendicular line (plumb line) from the horizon of the basement membrane of the adjacent mucosa to the deepest point of tumor invasion and recorded in millimeters. Perineural invasion (PNI) was considered positive when nerve involvement was present irrespective of being intratumoral/extratumoral and/or focal/multifocal. Lymphovascular invasion (LVI) was defined as the presence of tumor epithelial cells within or attached to the vascular endothelial lining. For documenting lymph node (LN) size, the maximum diameter of the lymph node dissected was selected.

The predictor variables in our study included site, depth of invasion, pattern of invasion, LVI (lymphovascular invasion), PNI (perineural invasion), grading, and lymph node size. The primary outcome was the influence of these factors on mandibular invasion, lymph node metastasis, and extracapsular spread. Overall survival was considered as the secondary outcome of the study.

### 2.4. Statistical Analysis

The statistical analysis was performed using IBM SPSS software, version 22.0 (IBM, Armonk, NY, USA).The receiver operating characteristic (ROC) curve and Youden’s J statistic were used to determine the optimal cut-off point for LN size and DOI. For LN size and DOI, patients were hence categorized into groups below and above the cut-off point. The Kaplan–Meir method was used to evaluate OS, and the log rank test was used to compare the survival among groups. Cox regression analysis (univariate and multivariate) was used to evaluate the hazard ratio and identify the possible factors influencing risk of death and disease. Regression analysis was also performed to evaluate the effect of predictor variables on primary outcome. A *p* value of <0.05 was considered statistically significant at 95% confidence interval.

## 3. Results

This evaluation included 162 patients with biopsy-proven oral squamous cell carcinoma (OSCC) treated between January 2013 and December 2021. (Table 1) The mean age of the patients was 54.78±6.4 years, with a male-to-female ratio of 1:0.18. The majority of tumors were located in the buccal mucosa. Tumor staging revealed that 38.9% of patients were classified as T3, while 53.7% were in the T4 stage. Pathological nodal positivity was identified in 42% of cases. Histologically, mandibular involvement was observed in 53.1% of patients. An aggressive pattern of invasion was present in 43.2% of cases, whereas 56.8% demonstrated a cohesive pattern.

With respect to mandibular invasiveness, 67 patients exhibited medullary involvement, and 19 had cortical involvement. Among the 86 patients with mandibular invasion, 56 had an infiltrative variant, and 30 demonstrated an erosive tumor front. Of the 56 patients with infiltrative tumor fronts, 50 showed an aggressive pattern of invasion, while six had a cohesive pattern. The aggressive pattern was more commonly associated with the infiltrative variant, which, in turn, was more frequently linked to bone marrow (medullary) involvement. Adjuvant therapy was administered in 55.5% of patients.

Among the factors influencing mandibular invasion, depth of invasion (DOI) and the pattern of invasion were significantly associated with clinical outcomes. Receiver operating characteristic (ROC) analysis using Youden’s J statistic identified a DOI cut-off value of 6.5 mm, which was predictive of outcomes with a sensitivity of 63%, specificity of 82%, and a Youden’s index of 0.43. On multivariate analysis, both DOI and the pattern of invasion emerged as independent risk factors for mandibular invasion (Table 2). With regard to lymph node metastasis, a DOI greater than 6.5 mm and an aggressive pattern of invasion were significantly associated. These two variables were also found to be independent predictors of lymph node metastasis (Table 3).

Extracapsular spread was significantly and independently influenced only by lymph node size. ROC analysis established a cut-off lymph node size of 2.95 cm, demonstrating a sensitivity of 71.2%, specificity of 87.9%, and a Youden’s index of 0.59 (Table 4).

The median overall survival (OS) in our study was 33 months (range: 29–36 months). Patients with an aggressive pattern of invasion had a median OS of 28 months, compared to 34 months in those with a cohesive pattern. Patients with lymph node metastasis had a median OS of 26 months, whereas node-negative patients had a median OS of 49 months (Table 5).

**Figure 1:**
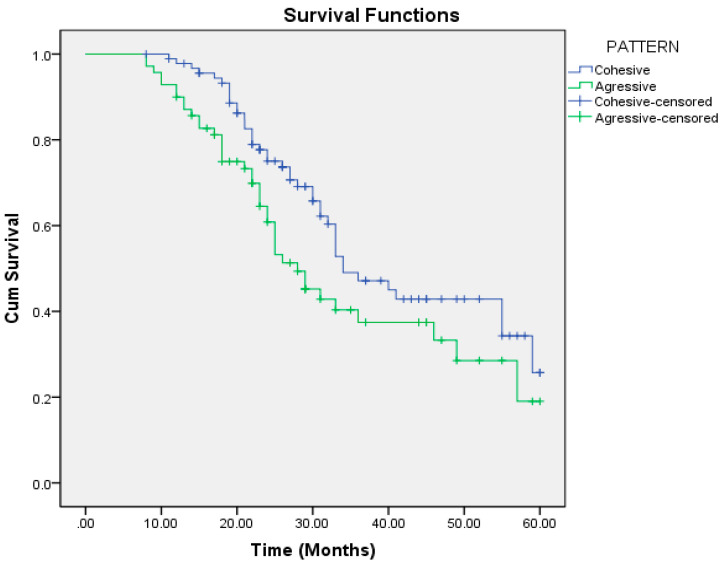
Pattern of invasion as a predictor of overall survival.

**Figure 2:**
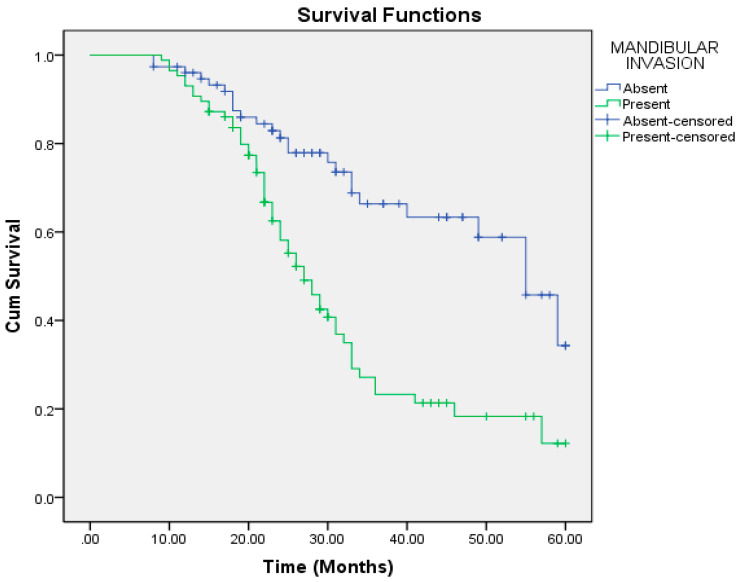
Mandibular invasion as a predictor of overall survival.

**Figure 3:**
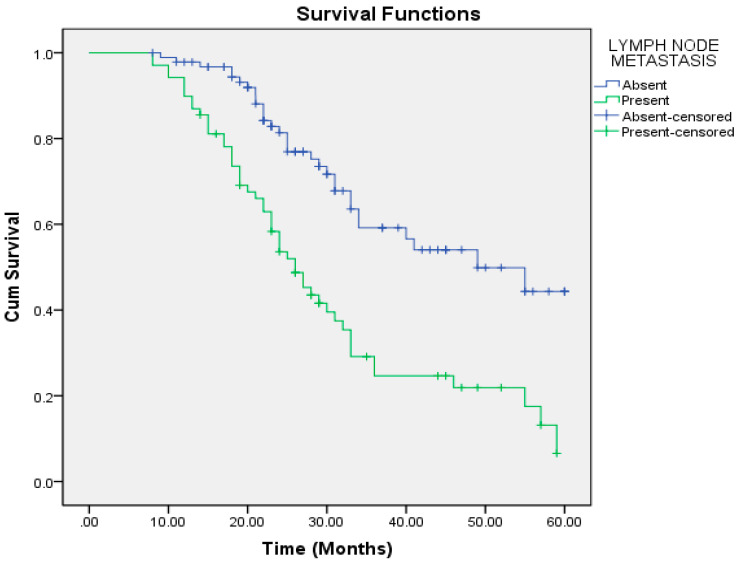
Lymph node metastasis as a predictor of overall survival.

**Figure 4:**
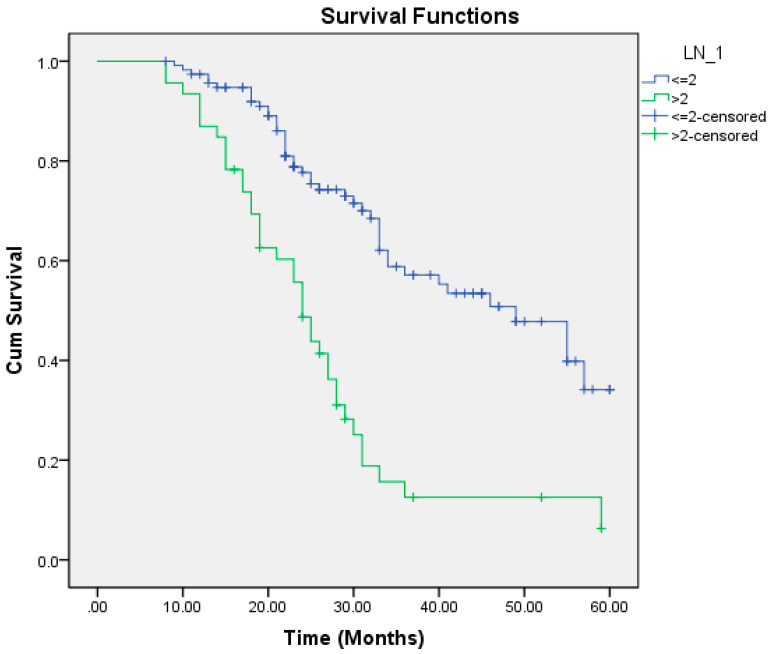
Lymph node size as a predictor of overall survival.

**Figure 5:**
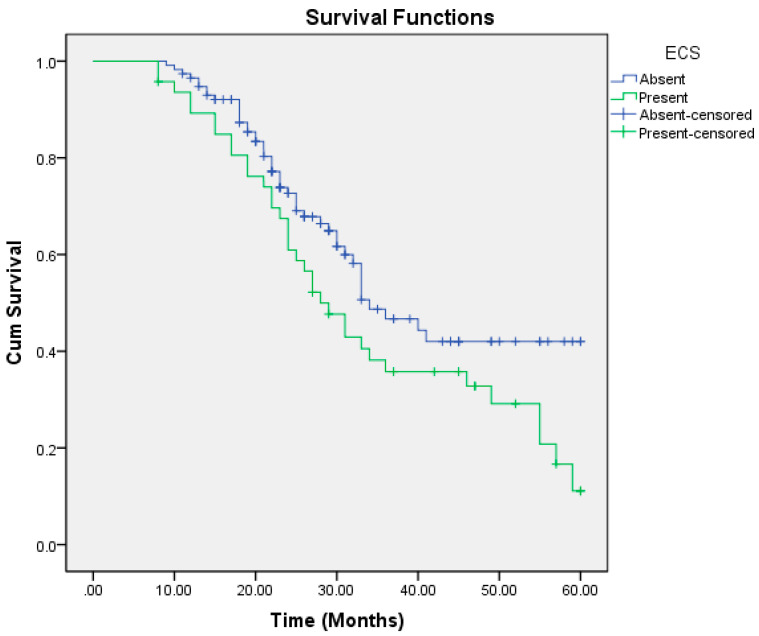
Extracapsular spread as a predictor of overall survival.

On univariate regression analysis, the following factors were significantly associated with poorer overall survival: aggressive pattern of invasion, T4a and T4b tumors, moderately and poorly differentiated tumors, nodal positivity, mandibular invasion, perineural and lymphovascular invasion, extracapsular spread (ECS), depth of invasion (DOI) >6.5 mm, and lymph node size >2.95 cm (Table 6). Multivariate regression analysis identified the pattern of invasion, mandibular invasion, lymph node metastasis, lymph node size, and ECS as independent risk factors for reduced overall survival (Table 7, Figure 1, Figure 2, Figure 3, Figure 4 and Figure 5).

In our study, patients with nodal metastasis had a median OS of 26 months compared to 49 months in those with a pathologically node-negative neck. There was no statistically significant difference in survival among different tumor subsites. The presence of ECS significantly impacted survival, with a median OS of 28 months compared to 34 months in its absence. Similarly, mandibular invasion reduced the median OS to 27 months, versus 55 months in patients without bony involvement. Lymphnode size greater than 2.95 cm was associated with a median OS of 24 months, compared to 49 months in nodes ≤2.95 cm.

## 4. Discussion

### 4.1. Interpretation

Two primary patterns of mandibular invasion have been described: the erosive and the infiltrative patterns. In the erosive pattern, the tumor advances with a broad front, separated from the bone by intervening connective tissue and active osteoclastic resorption—referred to as the osteoclast-dependent phase. In contrast, the infiltrative pattern is characterized by the absence of an intervening connective tissue layer, minimal osteoclastic activity, and the presence of tumor cell islands and projections extending directly into the medullary bone.

A third, mixed pattern of invasion may also be observed wherein tumors transition from an osteoclast-dependent to an osteoclast-independent phase, depending on the extent of invasion. The pattern of mandibular invasion is also influenced by both the depth and width of tumor infiltration, with the likelihood of bone invasion increasing proportionally with greater depth and width [18,19,20,21]. A 2002 systematic review reported that mandibular invasion is associated with poor overall survival (OS). Specifically, medullary invasion was linked to significantly worse OS, whereas cortical invasion showed no clear correlation with survival outcomes. Additionally, medullary involvement was associated with reduced disease-specific survival. Importantly, invasion of the medullary portion of the mandible was identified as a poor prognostic factor, regardless of tumor size [12,22].

The pattern of invasion (POI) has been identified as a potential indicator of lymph node metastasis [3,23,24]. In a study conducted by Chatterjee et al., 47 out of 48 patients with confirmed lymphatic metastasis exhibited an aggressive invasion pattern. Their findings further established POI as an independent risk factor for cervical lymph node involvement [3]. However, conflicting evidence exists, as some studies have reported no significant correlation between POI and lymphatic spread [25,26]. Mair et al. demonstrated a strong association between extracapsular spread (ECS) and specific tumor characteristics, including a depth of invasion (DOI) exceeding 5 mm, metastatic lymph nodes larger than 15 mm, and the presence of multiple involved neck nodes [5]. To refine ECS risk stratification, Lewis et al. proposed a grading system: **Grade 0** (tumor confined within the lymph node), **Grade 1** (tumor extending to the nodal capsule with capsular thickening), **Grade 2** (perinodal extension ≤1 mm beyond the capsule), **Grade 3** (extension >1 mm beyond the capsule), and **Grade 4** (soft tissue metastasis with no residual nodal tissue). While Grade 4 ECS correlated with worse clinical outcomes, its prognostic impact was not independent of other variables [27]. The mechanism of ECS involves progressive tumor infiltration: metastatic cells initially invade the subcapsular sinuses, followed by interfollicular and medullary sinuses, ultimately disrupting the nodal architecture and breaching the capsule. In larger nodes, mechanical expansion of the tumor mass may precipitate ECS, whereas in smaller nodes, tumor emboli lodged within the capsular sinuses can cause focal disruption, leading to extracapsular extension [28]. Toker [29] classified carcinomatous growth within cervical lymph nodes into four distinct patterns:

1. **Subcapsular Sinus Invasion:** Tumor cells initially proliferate in the subcapsular sinus, progressively replacing the nodal architecture. Extracapsular spread (ECS) arises either through direct capsular penetration or via tumor emboli trapped in adjacent capsular and juxtacapsular lymphatics.
2. **Lymphatic Sinus Infiltration:** Widespread tumor infiltration of the lymphatic sinuses occurs while sparing germinal centers and trabeculae. ECS may develop through mechanisms similar to those described above.
3. **Concurrent Intra- and Extranodal Proliferation:** Tumor cells exhibit simultaneous and proportional growth both inside the lymph node and in surrounding tissues.
4. **Extranodal Embolic Growth:** Tumor emboli proliferate without significant intranodal involvement, potentially leading to early ECS in the disease course.

Extracapsular spread (ECS) is influenced by a constellation of clinicopathological variables, including advanced nodal stage, metastatic lymph node dimensions, primary tumor location, histopathological characteristics, tumor thickness, multiplicity of involved nodes, midline-crossing tumors, and contralateral cervical metastases [4,28]. A pronounced correlation exists between nodal staging and ECS prevalence, with documented rates of 35% in N1 disease, 55% in N2, and 74% in N3 classifications. Notably, ECS incidence exhibits a size-dependent escalation: 60–100% in nodes exceeding 3 cm, 39–59% in nodes measuring 1–3 cm, and a modest 23% in subcentimetric nodal metastases [4]. The therapeutic approach to mandibular involvement in oral carcinoma is dictated by multifactorial considerations. Key determinants of resection strategy encompass tumor dimensions, depth of soft tissue infiltration, dentition status, and the extent of osseous invasion [20]. Neoplastic infiltration typically initiates at the tumor–mandible interface, with an erosive invasion pattern often warranting marginal resection to achieve oncological clearance [27].

### 4.2. Limitations

1. Prior radiotherapy and secondary tumors exhibit different POI, which was not taken into consideration in our study.
2. Several other factors like comorbidities, lifestyle, and patient physical status might impact survival. Further studies are necessary to evaluate the combined effects of each of these factors on survival.
3. In our study, the pattern of invasion was determined histologically. Various imaging modalities have been shown to determine the pattern of invasion, comparison of which was not performed in our study.
4. Smaller sample size and retrospective nature of the study.

## 5. Conclusions

Aggressive pattern of invasion and depth of invasion are independent risk factors for cervical lymph node metastasis and mandibular invasion. The only independent risk factor for extracapsular spread is lymph node size. Aggressive tumors preferentially show an infiltrative pattern of invasion into the mandible, necessitating segmental resection. Lymph node metastasis and nodal size, pattern of invasion, mandibular invasion, and extracapsular spread are independent risk factors affecting overall survival.

Further research and clinical trials are necessary in this regard for better description and identification of risk factors for early cervical metastasis and bone invasion.

Clinical Implication: The current study has elaborated the causative and predictor factors for lymph node metastasis, extracapsular spread, and mandibular invasion. Though individual studies exist, a thorough comprehensive study is lacking. Currently, only the primary site and depth of invasion are considered important prognostic factors for lymph node metastasis. Future studies should consider biologic behavior of the tumor. ECS hampers survival. Our study has shown that only the size of the metastatic lymph node is an independent risk factor, not the biologic behavior of the primary tumor. Tumor biology is an independent risk factor for mandibular invasion, necessitating aggressive management in cases of mandible involvement.

## Figures and Tables

**Table 1:** Study characteristics.

Characteristics	Frequency		
	No. of Patients		Percentage
Mean Age M:F ratio	54.78 ± 6.4 years 1:0.18		
Site of primary tumor	Buccal mucosa	40	24.70%
	Tongue	33	20.40%
	Floor of mouth	35	21.60%
	Mandibular alveolus	32	19.80%
	Retro molar trigone	22	13.60%
pT stage:	T2	12	7.4%
	T3	63	38.90%
	T4a	76	46.90%
	T4b	11	6.8%
pN stage:	N0	94	58.02%
	N+	68	42%
**Mandibular Invasion:** **Histopathology**	No involvement	76	46.9%
	Cortical involvement	19	11.73%
	Medullary involvement	67	41.40%
	Erosive	30	34.88%
	Infiltrative	56	65.11%
**Grading.**	Well	72	44.40%
	Moderate	47	29.0%
	Poor	43	26.5%
**LVI**	Absent	101	62.3%
	Present	61	37.7%
**PNI**	Absent	115	71%
	Present	47	29%
**Pattern of invasion**	Cohesive	92	56.8%
	Aggressive	70	43.2%
**DOI (mm)**	≤6.5	95	58.6%
	>6.5	67	41.4%
**ECS**	Absent	122	75.3%
	Present	40	24.7%
**Lymph node size (cm)**	≤2.95	116	71.6%
	>2.95	46	28.4%
**Mandibulectomy:**	Marginal	42	25.9%
	Segmental	120	74.1%
**Management of neck:**	Ipsilateral SND	130	80.24%
	Ipsilateral MRND	28	17.3%
	Ipsilateral MRND + contralateral SND	4	2.5%
**Adjuvant treatment:**	No adjuvant therapy	72	44.4%
	Radiotherapy	70	43.2%
	Chemoradiation	20	12.3%

LVI: lymphovascular invasion, PNI: perineural invasion, ECS: extracapsular spread, DOI: depth of invasion, SND: selective neck dissection, MRND: modified radical neck dissection.

**Table 2:** Factors influencing mandibular invasion.

Univariate Regression	OR	95% C.I.		*p* Value			OR	95% C.I		*p* Value
		Lower	Upper					Lower	Upper	
BM					**multivariate regression**	Site	0.830	0.639	1.079	0.165
FOM	2.746	0.782	9.643	0.115		size	0.940	0.537	1.645	0.828
Mn alveolus	1.701	0.501	5.782	0.395		DOI	2.810	1.200	6.580	0.017
RMT	1.266	0.295	5.439	0.751		GRADING	1.353	0.854	2.141	0.197
TONGUE	0.359	0.101	1.276	0.114		LVI	0.865	0.273	2.738	0.805
pT 2						PNI	2.162	0.607	7.706	0.234
pT 3	1.140	0.199	6.544	0.883		PATTERN	8.870	3.825	20.570	0.000
pT4	0.788	0.141	4.409	0.787		LN size	1.907	0.573	6.352	0.293
DOI ≤ 6.5										
DOI > 6.5 mm	3.273	1.283	8.348	0.013						
Well differentiated										
Moderate	0.702	0.253	1.946	0.496						
Poor	1.843	0.700	4.853	0.216						
LVI absent										
LVI present	0.810	0.237	2.772	0.738						
PNI absent										
PNI present	2.955	0.738	11.830	0.126						
Cohesive pattern										
Aggressive pattern	10.748	4.222	27.357	<0.001						
LN size ≤ 2.95										
LN size > 2.95 cm	3.544	0.917	13.705	0.067						

**Table 3:** Factors influencing lymph node metastasis.

Univariate Regression	OR	95% C.I.		*p* Value			OR	95% C.I.		*p* Value
		Lower	Upper					Lower	Upper	
BM					**multivariate regression**	Site	0.804	0.604	1.071	0.136
FOM	1.586	0.431	5.842	0.488		pT	1.525	0.797	2.917	0.203
Mn alveolus	0.767	0.199	2.956	0.700		DOI	6.486	2.453	17.144	<0.001
Tongue	0.916	0.159	5.288	0.922		GRADING	1.455	0.860	2.461	0.162
Site_1(4)	0.492	0.139	1.743	0.272		LVI	2.156	0.591	7.864	0.245
pT2				0.753		PNI	1.419	0.326	6.173	0.641
pT3	0.946	0.131	6.827	0.956		PATTERN	11.448	4.414	29.693	<0.001
pT4a	1.504	0.223	10.145	0.675						
pT4b	2.319	0.138	38.897	0.559						
DOI ≤ 6.5										
DOI > 6.5	7.155	2.547	20.099	<0.001						
Well differentiated										
Moderate	1.866	0.635	5.483	0.257						
Poor	1.993	0.682	5.823	0.207						
LVI absent										
LVI present	2.109	0.569	7.820	0.265						
PNI absent										
PNI present	1.475	0.324	6.711	0.615						
Cohesive pattern										
Aggressive pattern	11.516	4.332	30.611	<0.001						

**Table 4:** Factors influencing extracapsular spread.

Univariate Regression	OR	95% C.I.		*p* Value	Multivariate Regression	OR	95% C.I.		*p* Value
		Lower	Upper				Lower	Upper	
BM					Site	0.318	0.073	1.383	0.127
FOM	0.371	0.091	1.510	0.166	pT	1.120	0.592	2.118	0.728
Mn alveolus	0.296	0.067	1.311	0.109	DOI	0.845	0.357	2.005	0.703
RMT	0.955	0.193	4.723	0.955	GRADING	0.858	0.533	1.381	0.528
Tongue	3.684	0.908	14.941	0.068	LVI	1.503	0.483	4.674	0.481
pT2					PNI	0.575	0.153	2.158	0.412
pT3	8.302	0.490	140.752	0.143	PATTERN	1.293	0.406	4.120	0.663
pT4a	9.276	0.558	154.324	0.120	LN size	19.152	5.827	62.946	<0.001
pT4b	5.540	0.217	141.512	0.300					
DOI ≤ 6.5									
DOI > 6.5	0.563	0.215	1.478	0.244					
Well differentiated									
Moderate	1.317	0.344	5.041	0.688					
Poor	0.977	0.338	2.829	0.966					
LVI absent									
LVI present	1.491	0.433	5.140	0.527					
PNI absent									
PNI present	0.703	0.165	3.003	0.634					
Cohesive pattern									
Aggressive pattern	1.689	0.471	6.053	0.421					
LN size ≤ 2.95									
LN size > 2.95	27.795	6.953	111.119	0.000					

**Table 5:** Mean and median overall survival of the study population.

Mean and Median Survival Time (in Months)								
PATTERN	Mean				Median			
	Estimate	Std. Error	95% Confidence Interval		Estimate	Std. Error	95% Confidence Interval	
			Lower Bound	Upper Bound			Lower Bound	Upper Bound
Cohesive	40.521	2.057	36.489	44.553	34.000	3.294	27.544	40.456
Aggressive	34.430	2.472	29.585	39.275	28.000	2.564	22.974	33.026
pT								
T2	47.932	5.165	37.808	58.056				
T3	38.072	2.559	33.057	43.086	34.000	8.772	16.808	51.192
T4a	37.629	2.344	33.034	42.223	33.000	0.581	31.862	34.138
T4b	26.364	3.718	19.077	33.651	24.000	2.752	18.605	29.395
GRADING								
Well	51.016	2.214	46.676	55.356				
Moderate	34.065	2.353	29.452	38.677	33.000	1.301	30.450	35.550
Poor	26.022	2.362	21.391	30.652	21.000	1.634	17.798	24.202
LVI								
Absent	41.337	2.117	37.188	45.487	41.000	8.703	23.943	58.057
Present	33.374	2.359	28.751	37.997	30.000	2.279	25.533	34.467
PNI								
Absent	40.710	1.959	36.871	44.549	40.000	6.489	27.281	52.719
Present	31.908	2.657	26.701	37.115	28.000	1.424	25.209	30.791
MANDIBULAR INVASION								
Absent	45.664	2.298	41.160	50.169	55.000	4.125	46.914	63.086
Present	31.279	1.879	27.596	34.962	27.000	1.845	23.383	30.617
LYMPH NODE METASTASIS								
Absent	44.089	2.146	39.883	48.296	49.000	9.114	31.136	66.864
Present	30.720	2.140	26.526	34.914	26.000	2.087	21.909	30.091
ECS								
Absent	40.082	2.066	36.032	44.132	34.000	3.486	27.168	40.832
Present	34.337	2.624	29.193	39.481	28.000	2.703	22.702	33.298
DOI_1								
≤6.5	44.898	2.160	40.664	49.132				
>6.5	30.123	1.989	26.225	34.020	26.000	2.237	21.615	30.385
LN_size								
≤2.95	42.952	1.878	39.271	46.633	49.000	6.286	36.680	61.320
>2.95	26.657	2.263	22.221	31.092	24.000	1.270	21.512	26.488
Site								
BM	35.459	2.904	29.767	41.151	30.000	3.839	22.475	37.525
FOM	39.326	3.498	32.469	46.182	49.000	14.004	21.553	76.447
Mandibular alveolus	38.319	4.182	30.122	46.515	36.000	8.472	19.395	52.605
RMT	36.857	3.409	30.176	43.538	33.000	2.455	28.188	37.812
TONGUE	39.126	3.635	32.002	46.250	34.000	6.270	21.711	46.289
Overall	37.918	1.604	34.774	41.063	33.000	1.982	29.116	36.884

**Table 6:** Univariate regression analysis of factors influencing survival.

PATTERN	Frequency	*p* Value	OR	Lower 95% CI	Upper 95% CI
Cohesive	92				
Aggressive	70	0.046	1.560	1.008	2.414
pT					
T2	12				
T3	63	0.063	3.915	0.928	16.511
T4a	76	0.048	4.207	1.014	17.451
T4b	11	0.004	9.407	2.074	42.659
GRADING					
Well	72				
Moderate	47	<0.001	3.902	1.983	7.680
Poor	43	<0.001	7.629	3.987	14.598
LVI					
Absent	101				
Present	61	0.019	1.688	1.091	2.610
PNI					
Absent	115				
Present	47	0.008	1.816	1.165	2.831
MANDIBULAR INVASION					
Absent	76				
Present	86	<0.001	8.467	4.234	16.934
LYMPH NODE METASTASIS					
Absent	94				
Present	68	<0.001	12.172	5.596	26.475
ECS					
Absent	122				
Present	40	<0.001	8.411	3.862	18.317
DOI					
≤6.5	95				
>6.5	67	<0.001	2.924	1.860	4.597
LN size					
≤2.95	116				
>2.95	46	<0.001	4.476	2.838	7.058
SITE					
BM	40				
FOM	35	0.478	0.793	0.419	1.503
Mandibular alveolus	32	0.901	0.959	0.493	1.864
RMT	22	0.832	0.928	0.464	1.856
TONGUE	33	0.514	0.798	0.405	1.571
Total	162				

**Table 7:** Multivariate regression analysis of factors influencing survival.

	OR	95.0% CI for Exp(B)		*p* Value
		Lower	Upper	
DOI	0.987	0.565	1.722	0.963
GRADING	1.284	0.889	1.853	0.182
LVI	0.834	0.392	1.776	0.638
PNI	0.730	0.337	1.581	0.425
pT	1.213	0.882	1.668	0.235
MANDIBULAR INVASION	2.274	1.006	5.144	0.048
ECS	2.762	1.106	6.898	0.030
PATTERN	2.065	1.152	3.703	0.015
LN size	1.903	1.037	3.491	0.038
LYMPHNODE METASTASIS	2.790	1.056	7.367	0.038

## Data Availability

The original contributions presented in this study are included in the article. Further inquiries can be directed to the corresponding author.
